# Supplementary material for: Pectoral Fin Anomalies in tbx5a Knockdown Zebrafish Embryos Related to the Cascade Effect of N-Cadherin and Extracellular Matrix Formation
Source: J Dev Biol. 2019 Jul 12;7(3):15. doi: 10.3390/jdb7030015 (PMC6787601; doi:10.3390/jdb7030015)
Supplement: Supplementary file 1 [file jdb-07-00015-s001.pdf]

Supplementary Materials

Validation of the efficacy, traumatic effect, and off-target effects of the *tbx5a* morpholino in this study

1. *Using the survival rate as an index to rule out off-target effects*
   We tested the traumatic effect by comparing the survival rates of mismatch *tbx5a*-MO and Danieau buffer with wild type embryos. A traumatic effect of the microinjection in the zebrafish embryo was observed in approximately 2%–2.5% at 24 hpf (hours post-fertilization), which increased to 4% at 36 hpf and 6%–8% at 48 hpf, and remained unchanged after 48 hpf.
   We tested the off-target effect of morpholino by comparing the survival rates of mismatch *tbx5a*-MO and Danieau buffer group. The survival rate showed no significance between the mismatch *tbx5*-MO and Danieau buffer group. This result implied that the off-target effect of *tbx5*-MO was negligible in this study.

Survival rates of *tbx5a* gene knockdown and control embryos

Tbx5a-MO (mismatch)

	24hpf	36hpf	48hpf	60hpf	72hpf	#of embryo treated
19.4ng *tbx5a*-MO-1	64.8%	63.0%	53.7%	48.1%	42.6%	54
19.4ng *tbx5a*-MO-2	74.1%	74.1%	65.0%	40.8%	33.6%	41
Danieau buffer	82.0%	80.0%	78.0%	78.0%	78.0%	50
mismatch *tbx5a*-MO	81.5%	80.0%	76.6%	76.6%	76.6%	43
Wild type	84.0%	84.0%	84.0%	84.0%	84.0%	50

Survival rate = survival embryos / # of embryos treated x 100%

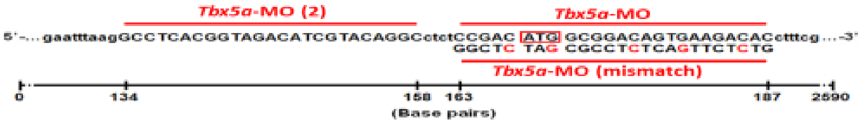

2. *The efficiency of the tbx5a-MO confirmed by Western blotting*

We used the TBX5a antibody with Western blot methodology to confirm the efficiency of *tbx5a*-MO to reduce the formation of TBX5 protein.

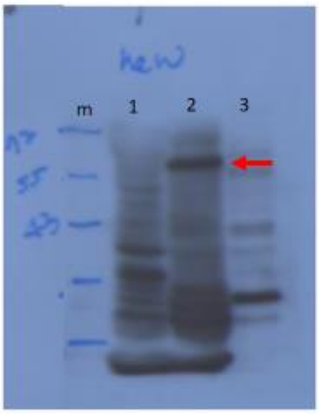

Red arrow: location of TBX5a at 58 KDa,

Lane1: *tbx5a*-MO zebrafish embryos at 48 hpf

Lane 2 : wild type zebrafish embryos at 48 hpf

Lane3 : contorl , M : marker

3. *Using the cardiac phenotype and heart function changes to rule out off-target effects*

   We used the (1) cardiac morphology and (2) heart rate as an index of cardiac function to verify the off-target effects of tbx5a-MO.

   The cardiac morphology of embryos microinjected with mismatch *tbx5a*-MO (n = 50) showed no cardiac defects compared with wild type embryos (n = 50). The rate of cardiac defects in embryos microinjected with *tbx5a*-MO was 52% at 24 hpf and increased to 64% at 36 hpf and 96% at 48 hpf. We found that tbx5a-Mo induced a high incidence of cardiac defects in zebrafish embryos, but embryos microinjected with *tbx5a*-mis morpholino did not show any cardiac defect. These results suggested that there were no off-target effects of tbx5a-MO leading to a cardiac defect.

   The heart rate of embryos microinjected with mismatch *tbx5a*-MO (n = 50) showed no differences compared with wild type embryos (n = 50), and it increased from 70 bpm (beat per minute) at 24 hpf to 110 bpm at 48 hpf. The heart rate of embryos microinjected with *tbx5a*-MO (n = 50) showed a significant reduction to 48 bpm at 24 hpf and increased to only approximately 85 bpm at 48 hpf. The cardiac output is directly related to the heart rate at the embryonic stage. We concluded that tbx5a-Mo induced a significant low cardiac output, but the cardiac output of embryos microinjected with the *tbx5a*-mis morpholino remained completely normal. These results suggested that there were no off-target effects of *tbx5a*-MO on cardiac function.

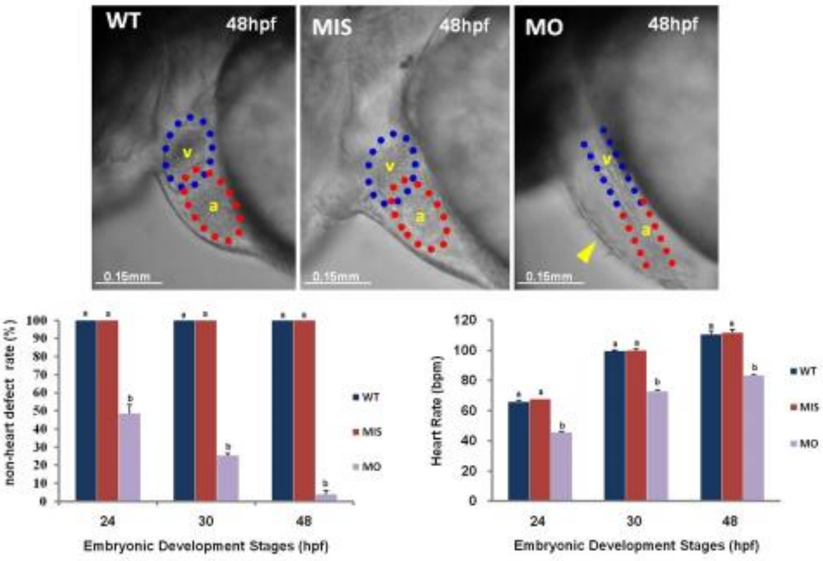

(WT: wild-type; MIS:*tbx5a*-mispair MO; MO:*tbx5a*-MO;a: atrium; v: ventricle ; arrow head :percardium effusion; hpf: hours post fertilization, n=50 embryos in each group)

4. *Using changes in pectoral fins to rule out off-target effects*

We used changes in pectoral fins as an index to rule out the off-target effects of *tbx5a*-MO.

The pectoral fins of embryos microinjected with mismatch *tbx5a*-MO (n = 50) showed no defects compared with wild type embryos (n = 50). The rate of pectoral defects in embryos microinjected with *tbx5a*-MO was approximately 85% at 24, 36, and 48 hpf. We found that *tbx5a*-Mo induced a high incidence of pectoral fin defects in zebrafish embryos, but embryos microinjected with the *tbx5a*-mis morpholino showed no pectoral fin defect. These results suggested that there were no off-target effects of *tbx5a*-MO interfering with development of the pectoral fin.

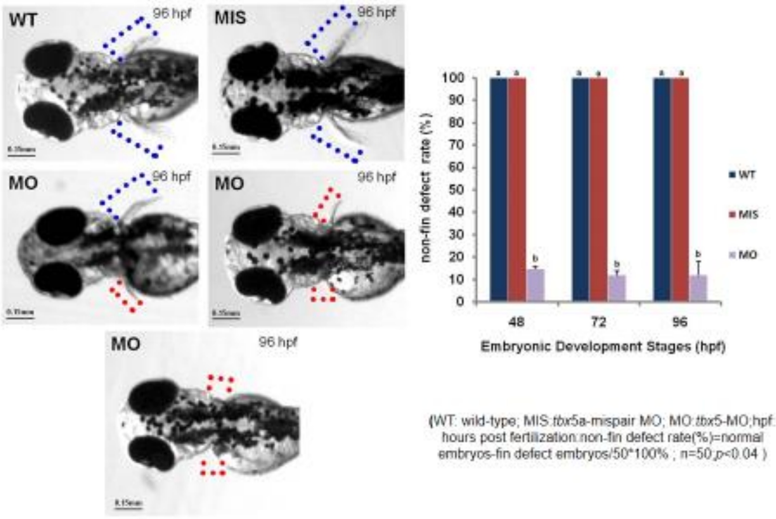

(WT: wild-type; MIS:*tbx5a*-mispair MO; MO:*tbx5*-MO;hpf: hours post fertilization:non-fin defect rate(%)=normal embryos-fin defect embryos/50*100% ; n=50;p<0.04)

5. *Using changes in trunk deformity to rule out off-target effects*

   We used changes in trunk deformity as an index to rule out the off-target effects of *tbx5a*-MO.

   The body trunk of embryos microinjected with mismatch *tbx5a*-MO (n = 50) showed no deformity compared with wild type embryos (n = 50). The trunk deformity rate in embryos microinjected with *tbx5a*-MO was approximately 75% at 24, 36, and 48 hpf. We found that *tbx5a*-Mo induced a high incidence of trunk deformity in zebrafish embryos, but embryos microinjected with the *tbx5a*-mis morpholino showed no pectoral fin defect. These results suggested that there were no off-target effects of *tbx5a*-MO leading to the induction of trunk deformity.

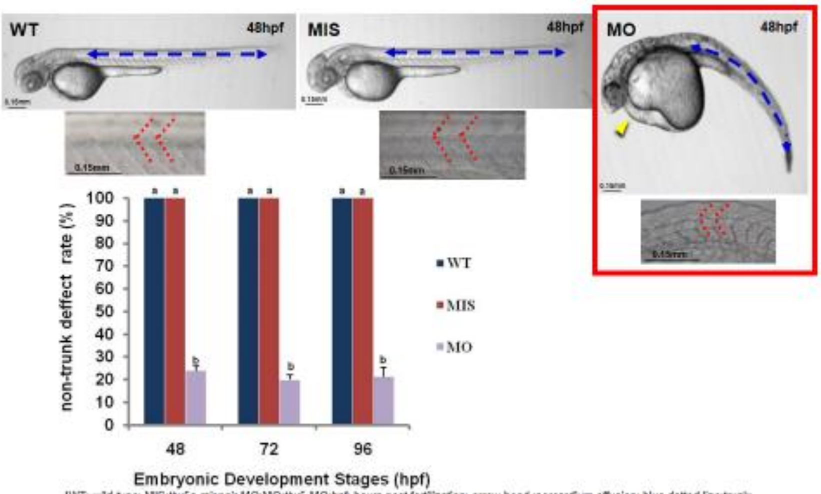

{WT: wild-type; MIS:*tbx5a*-mispair MO;MO:*tbx5*-MO;hpf: hours post fertilization; arrow head :percardium effusion; blue dotted line:trunk; red dotted line: somite; non-trunk defect rate(%)=normal embryos -trunk defect embryos /50*100%;n=50;p<0.05)
